# Healthcare personnel’s clinical decision-making competence in care for acutely ill older adults in home care: a cross-sectional study

**DOI:** 10.1186/s12912-026-04380-x

**Published:** 2026-02-04

**Authors:** Evy Gangstø Steinseide, Stein Erik Fæø, Sidsel Ellingsen, Milada Cvancarova Småstuen, Trine Oksholm

**Affiliations:** 1 Faculty of Health Sciences, VID Specialized University, Ulriksdal 10, Bergen, 5009 Norway; 2 Department of Nursing and Health Promotion, Faculty of Health Sciences, Oslo Metropolitan University, Pilestredet 32, Oslo, 0166 Norway; 3 The Dignity Centre, Ulriksdal 10, Bergen, 5009 Norway

**Keywords:** Home care services, Cross-sectional, Clinical decision-making, Older adults, Municipal health services, Survey, Primary health care, Aged, Clinical competence, Clinical assessment

## Abstract

**Background:**

With the increasingly aging population and a larger proportion of older adults living at home, healthcare personnel require well-developed clinical assessment skills and decision-making competence to detect and follow up on acute illnesses in older adults. A lack of such competence can threaten patient safety and must be identified. This study aimed to describe the level of clinical decision-making competence in Norwegian home care when healthcare personnel are caring for acutely ill older adults, and to explore whether selected background factors are associated with competence levels.

**Methods:**

A cross-sectional study was conducted among healthcare personnel in home care. Data were collected from 177 nurses, healthcare workers, and assistants using the Ms. Olsen competence test, which measures clinical decision-making. Descriptive statistics were computed, including the sum score of clinical competence, along with multiple regression analyses to examine associations with selected background variables.

**Results:**

Clinical decision-making competence was below the desirable cut-off for a large proportion of respondents across all groups, with 23.6% of nurses, 12.0% of healthcare workers, and 7.9% of assistants scoring above the cut-off. There was a particularly low score for correctly identifying diffuse symptoms. The results indicated uncertainty regarding which level of care to contact when a older adult’s health deteriorated. Factors associated with decision-making competence included the level of health education (*p* < 0.001) and the use of the ABCDE approach (*p* < 0.003).

**Conclusion:**

Clinical decision-making competence in Norwegian home care has room for improvement. There seems to be a need to enhance the competence of healthcare personnel to ensure that older adults receive the right help at the appropriate level of care. Uncertainty about what actions to take indicates a need for clearer guidelines regarding interventions in response to changes in conditions.

**Supplementary Information:**

The online version contains supplementary material available at 10.1186/s12912-026-04380-x.

## Introduction

Healthcare personnel (HCPs) worldwide are responsible for identifying changes in their patients’ health conditions and making decisions on how to follow up on these observations. Clinical decision-making is related to the mechanism of clinical reasoning, in which HCPs make professional judgements based on intuition, knowledge, and professional experience [1].

Clinical decision-making is an essential skill that ensures patient safety [2]. In Western countries, the number of older adults is rapidly increasing [3], and in Norway, there is a trend for older adults to live at home for as long as possible, driven by political objectives, higher life expectancy, and, in general, a healthier population [4]. Older patients tend to have poorer outcomes and are more vulnerable to deterioration from minor triggers [5]. They often present with diffuse symptoms of acute illness that are harder to detect [6]. In light of the challenges associated with the patient group and the demographic changes, a health system that supports and cares for older individuals living at home is needed [3].

Given the growing number of older adults at risk of deteriorating while living at home, there is a need to examine the level of HCPs’ clinical decision-making competence.

## Background

### The older patient group

Patient safety is a global priority, and the inability to identify deteriorating health conditions in older patients poses a potential threat, with possible consequences including missed care or, in the worst cases, failure to rescue [7]. Older patients constitute a vulnerable group prone to functional decline [8]. Aging is associated with increased frailty status, resulting in poor health due to compounded age-related deficits [9]. This vulnerability is often accompanied by atypical symptom presentations, which include nonspecific and subtle signs of general functional decline, making them less clinically obvious and harder to detect [6]. Accurate assessment of a patient’s status is regarded as highly important and plays a fundamental role in HCPs’ clinical decision-making [10].

### Support systems and tools

Several systems and tools have been developed for structured patient assessment to assist HCPs in their evaluations and clinical decision-making. The most commonly used tools in Norway include ABCDE, PEHA, NEWS2, and ISBAR (Table 1). Several of these are recommended in the national guidelines for identifying acute illness in Norway [11]. Additionally, measuring vital signs is essential for monitoring a patient’s condition and serves as the basis for many of these tools [12]. To our knowledge, there are no studies investigating how the use of these support systems and tools is associated with clinical decision-making competence in assessing older adults in municipalities.

**Table 1:** Description of frequently used support systems and tools

Support system or tool	Meaning	Description
Measuring vital signs	-	The most common vital signs used to assess patients are blood pressure, pulse rate, oxygen saturation, respiratory rate, body temperature, and level of consciousness [12, 13].
ABCDE	Airway, breathing, circulation, disability, exposure	A systematic approach that is used to promptly evaluate and manage patients with life-threatening conditions by prioritizing airway, breathing, circulation, disability, and exposure [14].
PEHA	Physical examination and health assessment	A systematic method for assessment, based on four fundamental pillars of examination methodology: inspect, percuss, palpate, and auscultate [15].
NEWS2	National Early Warning Score 2	An aggregated scoring system, where the six physiological vital signs give scores depending on how far from normal status they are. The total score indicates the patient’s clinical status and the need for follow-up [16].
ISBAR	Introduction, situation, background, assessment, recommendations	A framework for communication, especially used in acute situations. It systematizes information, making it structured, focused, and concise [17].

### Home care context in Norway

Norwegian home care is staffed by Registered Nurses (RNs) with at least a three-year bachelor’s degree in nursing from a university or college, authorized healthcare workers with a minimum of three years of health education at the high school level, or assistants without formal health education [18, 19]. The HCPs collaborate on all tasks and patient follow-up, with RNs taking on more specialized responsibilities while healthcare workers and assistants provide more practical support. However, given the rising demand for specialized care, the distinct roles of these professionals are becoming increasingly important [18, 19], as there has been a shift in home care, with municipalities now offering more specialized care and assuming greater responsibility for patients who are more ill [20].

However, resources in home care are limited, and HCPs often experience time pressure and insufficient time to follow up on the patient’s condition [21, 22]. As patients in home care have a greater level of illness than in previous years, HCPs must perform more advanced clinical assessments [23]. RNs play a key role in home care by providing care independently and frequently being the first to detect signs of deterioration in patients’ health [24]. Required competencies in home care include the ability to perform clinical assessments, think critically, and make informed decisions based on those assessments [25].

### Measuring clinical decision-making competence

Research has shown that HCPs differ in their ability to identify signs of deterioration, and vital signs are measured infrequently, across all roles, including assistants, healthcare workers, and RNs [26]. To identify learning needs and areas for professional development in home care, measuring competency is essential [27]. Previous studies have shown that HCPs working in municipalities have lower competence than expected, both in general competence measurements [28] and specifically in clinical decision-making [29, 30]. Bing-Jonsson et al. [28] explored the general competence in community elderly care among 1,016 HCPs in nursing homes and home care in Norway, revealing that while such competence exists, it is insufficient, with HCPs in home care scoring lower than those in nursing homes. The Ms. Olsen instrument was developed to measure clinical decision-making competence in municipalities [31]; It has been used in research in Norway and Finland [29–31]. Hopøy et al. [29] used it in nursing homes and reported varying competence among the HCPs. Vikström-Dahl et al. [30] used the Ms. Olsen test to explore clinical competence and decision-making skills in nursing homes in Finland where a cross-sectional study of 337 HCPs revealed concerning limitations in clinical competence. However, the Ms. Olsen test has not been explicitly used in studies involving HCPs in Norwegian home care, and the clinical decision-making competence of various groups of HCPs in this setting has yet to be thoroughly explored. Understanding this competence is crucial, as all groups of HCPs interact with fragile older individuals at risk of deteriorating health.

### Aim

This study aimed to explore clinical decision-making competence among RNs, healthcare workers, and assistants in Norwegian home care when assessing older patients’ acute illnesses.

Research question 1: What is the level of clinical decision-making competence among HCPs in Norwegian home care?

Research question 2: What are the possible associations between clinical decision-making competence and preselected background variables?

## Methods

### Study design

This was a cross-sectional study.

### Study setting and participants

The study was conducted in home care departments across five Norwegian municipalities from April 2024 to September 2024. These municipalities were selected for their geographical and demographical diversity, with populations ranging from a few thousand to over 150,000 (Table 2). Some rural areas are over two hours from hospitals, facing geographical challenges, such as ferry crossings and long driving distances. In contrast, more urban areas are within walking distance of nearby hospitals. The municipalities had different organizational structures, depending on whether HCPs worked in separate teams or were mixed. The geographical and demographic diversity is intentionally selected to serve as the best possible representation of Norwegian HCPs working with older adults. A total of 427 HCPs were invited to participate, with 177 responding (44% response rate). The sample included RNs, healthcare workers, and assistants working directly in patient-facing roles, regardless of whether they held permanent, temporary, or occasional positions. Based on a formula of 15 observations per independent variable (*n* = 10) planned for multiple regression analysis, the sample size of at least 150 respondents was deemed necessary.

**Table 2:** Respondent characteristics

	All *n* (%) 177 (100)		RNs *n* (%) 72 (40.68)		Healthcare workers *n* (%) 67 (37.85)		Assistants *n* (%) 38 (21.47)	
	*n*	(%) or mean ± SD	*n*	(%) or mean ± SD	*n*	(%) or mean ± SD	*n*	(%) or mean ± SD
Total number of participants	177	100	72	40.68	67	37.85	38	21.47
Gender								
Male Female	25 152	14.1 85.9	8 64	11.1 88.9	10 57	14.9 85.1	7 31	18.4 81.6
Age		38.8 ± 13.7 (range: 19–66)		38.4 (± 11.6) (range: 22–65)		44.6 (± 14.5) (range: 20–66)		29.8 (± 10.8) (range: 19–61)
Employment percentage								
0% 1–50% 51–75% 76–100%	24 11 15 127	13.5 6.2 8.5 71.8	1 2 5 64	1.4 2.8 6.9 88.9	4 7 7 49	6.0 10.5 10.4 73.1	19 2 3 14	50.0 5.3 7.9 36.8
Type of employment								
Permanent Temporary Occasionally	142 16 19	80.3 9.0 10.7	68 3 1	94.4 4.2 1.4	58 5 4	86.6 7.4 6.0	16 8 14	42.1 21.1 36.8
Number of years since last finished healthcare-related education		8.9 ± 10.2 (range: 0–39)		10 ± 9.6 (range: 0–37)		11.9 ± 11.2 (range: 0–39)		1.1 ± 4.0 (range: 0–20)
Number of years working in healthcare		13.5 ± 10.5 (range:1–44)		15.3 ± 9.8 (range: 1–40)		16.6 ± 11.4 (range: 1–44)		4.9 ± 4.3 (range: 1–20)
Language								
Norwegian as their first language Not Norwegian as a first language	147 30	83.1 16.9	65 7	90.3 9.7	53 14	79.1 20.9	29 9	76.3 23.7
Size of municipality								
Under 5000 citizens Between 5000 and 50 000 citizens Between 50 000 and 150 000 citizens Over 150 000 citizens	31 28 48 70	17.5 15.8 27.1 39.6	12 8 22 30	16.7 11.0 30.6 41.7	12 13 16 26	17.9 19.4 23.9 38.8	7 7 10 14	18.4 18.4 26.3 36.9

Abbreviations: SD – standard deviation

### Data collection

Five municipalities agreed to participate in this study when contacted, each appointing a contact person, who assisted in the recruitment process. During recruitment, the contact person distributed brochures containing study information, including links and QR codes, to potential respondents via email and in meetings. All participants received written information about the project before participating. They were informed about the project’s purpose, that participation was voluntary, anonymous, and that they could withdraw at any time until the survey was completed. After that, there was no possibility of identifying the answers due to anonymity. Informed consent to participate in the study was collected from all respondents. The questionnaire was web-based, using the digital platform *Nettskjema*, and could be completed on computers or smartphones without tracking IP addresses, to ensure privacy. When accessing the digital questionnaire, the participants had to actively select “yes” to participate; selecting “no” would close the questionnaire without further prompts.

The questionnaire collected information about background variables, including age, gender, education, employment type and percentage, years of experience, and first language (Table 2). It also collected background information about their “evaluation of own competence”, their “need for more competence”, “training in clinical assessments” or “specific support systems or tools, and “use of support systems or tools” (Table 3).

**Table 3:** Self-evaluation of competence, training and use of support systems

	All *n* (%) 177(100)		RNs *n* (%) 72 (40.7)		Healthcare workers *n* (%) 67 (37.9)		Assistants *n* (%) 38 (21.5)	
	*n*	%	*n*	%	*n*	%	*n*	%
Evaluation of own competence								
Experience they have enough competence Experience they do not have enough competence	117 60	66.1 33.9	50 22	69.4 30.6	44 23	65.7 34.3	23 15	60.5 39.5
Need for more competence								
Experience a need for more Do not experience the need for more	150 27	84.7 15.3	60 12	83.3 16.7	54 13	80.6 19.4	36 2	94.7 5.3
Training in clinical assessments*								
Yes No	93 84	52.5 47.5	38 34	52.8 47.2	39 28	58.2 41.8	16 22	42.1 57.9
Training in specific support systems or tools*								
No training Measuring vital signs ABCDE assessment PEHA News2 ISBAR communication tool	27 108 90 29 125 85	15.3 61.0 50.8 16.4 70.6 48.0	12 41 46 22 54 50	16.7 56.9 63.9 30.6 75.0 69.4	4 47 29 6 52 26	6.0 70.1 43.3 9.0 77.6 38.8	11 20 15 1 19 9	28.9 52.6 39.5 2.6 50.0 23.7
Use of support systems or tools								
None Measures vital signs ABCDE assessments PEHA NEWS2 ISBAR communication tool	10 141 91 20 136 79	5.6 79.7 51.4 11.3 76.8 44.6	2 66 55 14 63 54	2,8 91,7 76,4 19,4 87,5 75,0	0 53 25 4 52 20	0.0 79.1 37.3 6.0 77.6 29.9	8 22 11 2 21 5	21.1 57.9 28.9 5.3 55.3 13.2

Abbreviations:

ABCDE: airway, breathing, circulation, disability, exposure

ISBAR: introduction, situation, background, assessment, recommendations

NEWS2: National Early Warning Score 2

PEHA: Physical examination and health assessment

RN: registered nurse

*last two years

### The Ms. Olsen test

To investigate clinical decision-making competence, we used the Ms. Olsen test, specifically designed to assess clinical decision-making as an outcome [31]. This test was developed for assessing RNs, healthcare workers, and assistants working with older people in municipalities. The validated Ms. Olsen test comprises 19 items (Supplementary Table 1). It begins with a hypothetical case, with the following description: “Ms. Olsen is 90 years old and generally weakened by age” [31]. Following this case, respondents are presented with various symptoms exhibited by Ms. Olsen and are prompted to select one of six possible actions. These options range from “no action at all” to “providing acute help in the hospital” (Supplementary Table 1). A predefined score sheet has been developed, listing correct answers for each item, with varying correct answers for RNs, healthcare workers, and assistants [32] (Supplementary Table 1). Each correct answer in the Ms. Olsen test is assigned one point, allowing for a maximum total score of 19 points. When the Ms. Olsen test was developed, cut-off scores indicative of clinically sound practice were also established [31]. Due to the differing responsibilities among professional groups, the cut-off scores vary between RNs and healthcare workers or assistants [31]. For RNs, the cut-off requires correct answers for two-thirds of the items, whereas for healthcare workers and assistants, a minimum of half of the items must be answered correctly.

The Ms. Olsen test is derived from the Nursing Older People Competence Evaluation Tool (NOP-CET) [31]. Psychometric evaluation of the NOP-CET demonstrated acceptable content and construct validity, reliability, precision, interpretability, acceptability, and feasibility [32]. The Ms. Olsen test shows good measurement properties for RNs, healthcare workers, and assistants [31].

### Data analysis

Statistical analysis was performed using IBM SPSS Statistics version 29.0.1.0 and Stata version 18.5. The characteristics of the respondents were summarized using descriptive statistics: categorical data were reported as frequencies and percentages, while continuous data were described with means and standard deviations. Missing data were not a concern, as completion of all questions was mandatory for respondents.

Total scores (sum scores) were calculated for the overall sample as well as for each professional group. The competence score calculated from the test was treated as a continuous variable and served as the main outcome (dependent variable) in all regression analyses.

A multiple linear regression analysis was employed to assess the strength of potential associations between the dependent variable (level of clinical decision-making competence) and selected background variables. To evaluate the strength of associations between the outcome (items from the Ms. Olsen questionnaire as the dependent variable) and selected predictive factors (independent variable), several multivariate linear regression models were fitted. The questionnaire items were modeled separately as dependent variables. Model fit was checked, and the assumptions of linear regression, including independence and normal distribution of residuals, were fulfilled. Normality of residuals was evaluated through visual inspection (Q–Q plots and histograms) and the Kolmogorov-Smirnov test. The results of the linear regression are presented as regression coefficients with 95% confidence intervals (CIs) and p-values. A p-value of < 0.05 was considered statistically significant. All tests were two-sided.

## Results

### Respondent characteristics

The sample consisted of 177 individuals, including 72 (40.68%) RNs, 67 (37.85%) healthcare workers, and 38 (21.47%) assistants (Table 2).

Approximately half (52.5%) of all respondents reported having received general training in clinical assessments within the past 2 years, and 84.7% indicated they had received training in specific support systems or tools (Table 3).

### Levels of competence

The analysis revealed that the level of competence was insufficient across all three groups. Only one in four RNs (23.6%) achieved the required cut-off score, with the number of correct answers ranging from 5 to 15 out of 19 questions (Supplementary Table 2). Among healthcare workers, only 12% exceeded the cut-off score, with correct answers varying from 1 to 12. For assistants, 7.9% surpassed the cut-off score, with total correct answers ranging from 1 to 10.

Due to the low competence scores, we conducted additional analyses to determine whether respondents provided answers that indicated a level of help for Ms. Olsen that exceeded the recommendations based on the original test criteria. Consequently, we expanded the correct answers to include responses reflecting a higher level of help than originally outlined (Supplementary 1) and recalculated the new sum score. For example, in item number 19, where Ms. Olsen “has short attention span and delusions,” the correct answer is to undertake nursing-related measures immediately. In the revised analysis, we also included alternatives where respondents had chosen to contact a general practitioner (GP) or sought acute help in a hospital as correct answers. These new analyses showed that 85% of RNs, 88% of healthcare workers, and 74% of assistants provided the recommended level of help or higher.

### Factors associated with healthcare personnel’s competence

Participants who utilized support systems and tools had significantly higher scores in the dependent variable of clinical decision-making competence (B = 2.7; 95% CI (-4.6 to -0.9)) (Table 4). Notably, the use of the support system ABCDE was statistically significantly associated with higher scores (B = 1.2; 95%CI (0.4 − 2.0)). When accounting for their professional education, healthcare workers scored approximately 5 points lower on the Ms. Olsen test compared to RNs (95% CI (-5.7 to -4.1)), while assistants scored about 6 points lower than RNs (95% CI (-6.7 to – 4.6)). The adjusted R-squared indicated that our model accounted for 65% of the variance in competence level. No statistically significant relationships were found between clinical decision-making competence and age, gender, no training in support systems or tools, or other variables related to the use of support systems or tools (measures vital signs, PEHA, NEWS2, and ISBAR communication tool).

**Table 4:** Regression analysis

Variable	All participants (*n* = 177)		
	B	95% CI	*p*
No training in support systems or tools	0.56	(-0.44 to 1.57)	0.270
No use of support systems or tools	-2.75	(-4.57 to -0.94)	**0.003**
Measures vital signs	0.00	(-0.91 to 0.92)	0.989
Uses ABCDE assessments	1.25	(0.43 to 2.08)	**0.003**
Uses PEHA	0.20	(-0.85 to 1.27)	0.702
Uses NEWS2	-0.51	(-1.43 to 0.40)	0.269
Uses ISBAR communication tool	-0.50	(-1.37 to 0.36)	0.252
Age	0.01	(-0.00 to 0.04)	0.146
Gender	-0.29	(-1.21 to 0.61)	0.524
Occupation			
Nurses (RNs)	Reference		
Healthcare workers	-4.98	(-5.79 to -4.17)	**< 0.001**
Assistants	-5.65	(-6.70 to -4.60)	**< 0.001**
Model fit	*R*^*2*^ = 0.671, Adjusted *R*^*2*^= 0.650, F = 30.71, *p* < 0.001		

Abbreviations: B, regression coefficient; CI, Confidence interval

Multiple regression analysis was also conducted for the following background variables: employment percentage, years since their last health-related education, years of experience, first language, size of municipality, self-assessed competence, and recently completed competence-enhancing measures or courses related to clinical assessment. After adjusting for education, none of these factors were statistically significantly associated with the level of clinical decision-making competence.

### Items with high and low correct responses

Substantial variations existed between the items on which respondents scored highly and those on which they scored low (Table 5). Items describing more diffuse symptoms exhibited lower rates of correct responses. Item number 16, which involved Ms. Olsen experiencing symptoms of changes in sight, hearing, speech, and comprehension, emerged as one of the most challenging items for all groups. Only 1.4% of the RNs, 4.5% of the healthcare workers, and none of the assistants answered correctly. Items 3 (Has irregular pulse increased to more than 20/min within last two days) and 19 (Has short attention span and delusions) also had low correct response rates across all groups (Table 5). Conversely, item number 14, which addressed symptoms of partial paralysis, a more overt acute change, had the highest rate of correct answers, with nearly all respondents answering correctly (RNs: 100%, healthcare workers: 95.5%, and assistants: 92.5%).

**Table 5:** Overview of correct answers per item

Item no.		Total share of correct answers (%)	Correct answers RNs (%)	Correct answers Healthcare workers (%)	Correct answers Assistants (%)
1	Has dyspnoea during rest within last two days	51.40	66.70	50.70	23.70
2	Coughs, has increased saliva and respiration frequency above 20/min	41.20	93.10	4.50	7.90
3	Has irregular pulse increased to more than 20/min within last two days	6.80	11.10	6.00	0
4	Has temperature above 38.5	41.80	83.30	16.40	7.90
5	Is substantially dehydrated	41.20	69.40	26.90	13.20
6	Skin has rash, wounds, is red or itchy	21.50	36.10	11.90	10.50
7	Has reduced appetite and food intake	62.10	56.90	67.20	63.20
8	Is not able to eat	40.10	81.90	11.90	10.50
9	Has pain and discomfort in mouth	27.10	47.20	16.40	7.90
10	Is incontinent of urine, stings when urinates	48.60	95.80	20.90	7.90
11	Has fresh blood in stool	57.10	59.70	62.70	42.10
12	Has increased needs to full-time care within last two days	33.90	63.90	11.90	15.80
13	Has fallen two times during previous week	41.20	81.90	14.90	10.50
14	Has symptoms of partial paralysis	96.60	100	95.50	92.10
15	Is more tired during the day	30.50	26.40	34.30	31.60
16	Has changes in sight, hearing, speech and comprehension	2.30	1.40	4.50	0
17	Has newly occurring chest pain	85.90	93.10	86.60	71.10
18	Has lost interest in keeping home in order, sleeps in chair instead of bed	10.70	0	20.90	13.20
19	Has short attention span and delusions	8.50	9.70	6.00	10.50

## Discussion

This study aimed to investigate the clinical decision-making competence of HCPs in Norwegian home care and explore possible associations between competence levels and selected background variables. The main finding is that clinical decision-making competence appears to be limited and has room for improvement.

The identification of insufficient clinical decision-making competence in this study is supported by findings from prior research conducted in nursing homes [30] and in community care more broadly [28, 29, 33]. The results on differences in clinical decision-making competence across professional groups were expected, given the varying levels of education and responsibility. RNs are expected to continuously develop their professional role and skills [24]. In contrast, the expectations regarding clinical skills among other professional groups remain ambiguous [35], despite evidence indicating that RNs and healthcare workers require similar competencies due to their responsibilities for making independent clinical decisions [34]. This independence is particularly crucial in home care settings, where HCPs typically operate autonomously and must continuously assess changes and respond to situations [24]. In the Norwegian context, there is an emerging recognition of the necessity for more systematic education and competency development across all professional groups in response to healthcare challenges [35]. The results of this study emphasize the need for systematic education and training for all professions to fulfill these expectations.

In our study, we found surprisingly minimal differences in the decision-making competence between healthcare workers and assistants. One possible explanation is that the assistants had received training, thereby equipping them with knowledge of appropriate actions—this has been a significant focus within patient safety programs [36]. There has been a notable shift in tasks for both healthcare workers and assistants [35]. Although assistants traditionally focus on delivering personal care, they are increasingly involved in assessments and patient monitoring [37, 38]. This shift may have led to assistants receiving more training. Another potential explanation for the minimal difference in competence between healthcare workers and assistants that we found in our study is that neither group views assessments as their primary task, often referring decision-making to RNs. This is supported by a study conducted by Craftman et al. [38], in which assistants expressed a preference for RNs to conduct assessments and make the decisions. However, some assistants in our study were health students, which may have affected their competence in clinical decision-making. Additionally, some possessed extensive experience and may have acquired clinical knowledge through various situations and reflective practice.

While we lack specific information about the reason for the low competence scores in this study, contextual factors may have influenced the results. Home care settings frequently report resource shortages, and the ability to develop competence is contingent upon leadership prioritization [39]. RNs in other studies have indicated that they face structural constraints due to limited resources and an emphasis on daily operations, which impedes their ability to maintain their skills and follow up with patients [21, 22]. The organization of home care practice has previously been found to affect HCPs’ assessment of clinical deterioration, possibly because detailed, pre-planned work schedules leave little room for independent decisions [26]. Furthermore, time constraints and staffing levels have been identified as barriers to further training for assistants [40]. The scarcity of resources necessitates prioritization, and our findings—which indicate that only approximately half of the respondents have received training in clinical assessments—suggest that training should be accorded higher priority.

Upon examining the level of clinical decision-making competence, our reanalysis with more expanded defined responses revealed that while HCPs do seek help, they often do not contact the appropriate level of care. For instance, many HCPs opt to contact GPs or the hospital rather than discuss their concerns with an RN or colleagues. This practice can lead to increased costs and a higher likelihood of unnecessary hospital admissions or transfers to GPs, with a risk of hospital-acquired infections, delirium, or adverse drug effects [5]. Variations have previously been reported in how HCPs respond to changes in patient status [26, 41]. Based on observations and focus group interviews with 30 nurses and healthcare workers, Strømme et al. [26] found that healthcare workers frequently expressed uncertainty regarding appropriate actions. They sometimes delayed their response and monitored patients or sought assistance from a GP when indications of patient deterioration arose. Meanwhile, assistants stated that they always contacted RNs. These findings align with the results of the present study and highlight a pervasive insecurity regarding which level of care to consult for assistance. It can be posited that an increased emphasis on resource utilization to build competence could enhance HCPs’ confidence in handling such situations, thereby optimizing resource allocation. For RNs to feel confident in the care they provide, it is essential to develop their clinical decision-making and reasoning skills, as these have been shown to facilitate more efficient and autonomous care [42]. This aligns with a Norwegian government white paper, which asserts that appropriate clinical decision-making competence can mitigate the overutilization of emergency medical services [43].

This study indicates that diffuse symptoms represent the most challenging aspects for HCPs to identify and address. Previous research has shown that HCPs often perceive diffuse symptoms as normal conditions [26]. This issue may be particularly relevant for older adults, a frail population characterized by varying health conditions and multiple diagnoses, which can complicate illness recognition [5]. The highest-scoring items we identified were typically symptoms with well-established guidelines after public education efforts, such as paralysis and stroke [44]. While guidelines can facilitate accurate decision-making, real-world challenges reveal gaps in practical application [45]. The scarcity of standardized guidelines for responding to clinical deterioration [26], may contribute to HCPs’ uncertainty regarding appropriate actions, as evidenced by our findings.

Our findings suggest that HCPs who did not utilize any support systems or tools at all scored lower than their counterparts who did. This indicates that the use of such systems is beneficial for assessments and serves as a basis for decision-making. The finding is supported by research showing that tools support and systematize observations, function as reminders, and guide communication [14, 46, 47]. In this study, the ABCDE approach emerged as the only support system that was statistically significantly correlated with clinical decision-making competence. A plausible explanation for this correlation is that the ABCDE approach is relatively straightforward and can be learned across all professional levels. It relies on observations, and the assessment can be conducted without equipment [14].

### Strengths and limitations

“Competence” is multifaceted, and the Ms. Olsen test measures participants’ answers to written clinical questions rather than providing an objective evaluation of their responses in real-life situations, which is a common limitation of surveys. It is likely that most HCPs would perform a more complex assessment than the response options in the survey permit. Additionally, the survey’s wording and the instruction to select only one answer may lead to uncertainty among respondents. However, the Ms. Olsen test has been validated and tested, and the correct answers have been established using an expert group [29, 48]. The additional analysis we did using expanded correct answers is not part of the validated Ms. Olsen, but it did give valuable information. However, the “correctness” of these answers may be contested by some due to the variability and evolving nature of guidelines. A notable strength of this test is that it is not based on self-evaluation [49], unlike traditional assessments of nursing competence [27].

This exploratory study investigated potential associations between preselected available variables and the outcomes, with variables selected based on prior research. It could be argued that other, more relevant variables may warrant investigation but were unavailable for this study.

The response rate was low, a common issue in studies of this nature, which limit the generalizability and precision of the statistical estimates. Surveys generally face challenges in obtaining respondents, and one consideration in conducting surveys is the profile of the responders. It is possible that the most confident individuals—or those who are interested in developing their competence—are the ones who respond, which may introduce selection bias. In this study, a lower proportion of assistants responded than the RNs and healthcare workers. The study utilized a convenience sample, which may have affected the results and limited the generalizability of the findings. However, the selection of respondents was consistent with the demographic characteristics of HCPs in Norwegian municipalities in several aspects, including age distribution, gender, origin, and part-time employment status [35, 50].

## Conclusions

The increasing number of older individuals living at home necessitates competent HCPs who can respond effectively to their needs. This study indicates that there is substantial room for improvement in clinical decision-making competence, underscoring the importance of implementing competence-enhancing measures across all professional groups. Results show that HCPs often take actions that exceed what is necessary, potentially leading to increased costs and unnecessary patient transfers. This uncertainty in managing such situations highlights the need for explicit guidelines. This necessity is further supported by the findings, which suggest that diffuse symptoms are more challenging to detect and address.

Further research is needed to understand why HCPs may not act in certain patient situations and what motivates them to act in other cases. Additionally, there remain unanswered questions regarding the utilization of support systems.

## Supplementary Information

Below is the link to the electronic supplementary material.

Supplementary Material 1

## Data Availability

The datasets generated and analyzed in this study are not publicly available due to limitations of ethical approval but are available from the corresponding author on reasonable request.

## Abbreviations

ABCDE: Airways Breathing Circulation Disability Exposure
GP: General practitioner
HCPs: Health care personnel
ISBAR: Identification Situation Background Assessment Recommendation
NEWS2: National Early Warning Score 2
PEHA: Physical examination and health assessment
RNs: Registered nurses
