# Supplementary material for: Healthcare personnel’s clinical decision-making competence in care for acutely ill older adults in home care: a cross-sectional study
Source: BMC Nurs. 2026 Feb 4;25:208. doi: 10.1186/s12912-026-04380-x (PMC12964621; doi:10.1186/s12912-026-04380-x)
Supplement: Supplementary file 1 — Supplementary Material 1 [file 12912_2026_4380_MOESM1_ESM.docx]

## Supplementary nr 1: Ms Olsen test

‘Ms. Olsen is 90 years old and generally weakened by age. Imagine that she develops the following symptoms. Please choose how you would respond when Ms. Olsen, your patient, develops the following symptoms. You may choose one option on each line’.

| Item no. | Statement | Correct response | | Correct response after expanding answers | | |
| --- | --- | --- | --- | --- | --- | --- |
|  |  | RNs | Healthcare workers and assistants | RNs | Healthcare workers and assistants | |
| 1 | Has dyspnoea during rest within last 2 days | 5* | 4 or 5 | 5 or 6 | 4, 5 or 6 | |
| 2 | Coughs, has increased saliva and respiration frequency above 20 breaths/min | 4 or 5 | 4 | 4, 5 or 6 | 4, 5 or 6 | |
| 3 | Has irregular pulse increased to more than 20 beats/min within last 2 days | 4 | 4 | 4, 5 or 6 | 4, 5 or 6 | |
| 4 | Has temperature above 38.5 | 4 or 5 | 4 | 4, 5 or 6 | 4, 5 or 6 | |
| 5 | Is substantially dehydrated | 4 or 5 | 4 | 4, 5 or 6 | 4, 5 or 6 | |
| 6 | Skin has rash, wounds, is red or itchy | 4 | 4 | 4, 5 or 6 | 4, 5 or 6 | |
| 7 | Has reduced appetite and food intake | 3 or 4 | 3 or 4 | 3, 4, 5 or 6 | 3, 4, 5 or 6 | |
| 8 | Is not able to eat | 4 or 5 | 4 | 4, 5 or 6 | 4, 5 or 6 | |
| 9 | Has pain and discomfort in mouth | 4 | 4 | 4, 5 or 6 | 4, 5 or 6 | |
| 10 | Is incontinent for urine, experiences stinging when urinating | 4 or 5 | 4 | 4, 5 or 6 | 4, 5 or 6 | |
| 11 | Has fresh blood in stool | 5 | 4 or 5 | 5 or 6 | 4, 5 or 6 | |
| 12 | Has increased needs to full-time care within last 2 days | 4 or 5 | 4 | 4, 5 or 6 | 4, 5 or 6 | |
| 13 | Has fallen two times during previous week | 4 or 5 | 4 | 4, 5 or 6 | 4, 5 or 6 | |
| 14 | Has symptoms of partial paralysis | 6 | 4 or 6 | 6 | 4, 5 or 6 | |
| 15 | Is more tired during the day | 5 | 4 or 5 | 5 or 6 | 4, 5 or 6 | |
| 16 | Has changes in sight, hearing, speech and comprehension | 4 | 4 | 4, 5 or 6 | 4, 5 or 6 | |
| 17 | Has newly occurring chest pain | 6 | 4 or 6 | 6 | | 4, 5 or 6 |
| 18 | Has lost interest in keeping home in order, sleeps in chair instead of bed | 6 | 4 or 6 | 6 | | 4, 5 or 6 |
| 19 | Has short attention span and delusions | 4 | 4 | 4, 5 or 6 | | 4, 5 or 6 |

*On each item, the respondents are given the following alternatives:

[1] no action required

[2] observe again the following day

[3] consult with an RN

[4] nursing-related measure required immediately

[5] have patient assessed by physician

[6] requires acute help in hospital

[7] I do not know

The respondents have one answer per item.

Correct response refers to correct response as developed in Ms Olsen test.

Correct response after expanding answer, refers to answers used in additional analyses where we want to know if the HCPs did do any measures at all, or if the patient didn’t get any help. Here we also included correct answers in higher measures than originally.

## Supplementary nr 2: Sum score for each profession

|  | Nurses (RNs) |  | Healthcare workers | | Assistants |  |
| --- | --- | --- | --- | --- | --- | --- |
| **Number of items correct** | ***n*** | **%** | ***n*** | **%** | ***n*** | **%** |
| 0 | 0 | 0 | 0 | 0 | 0 | 0 |
| 1 | 0 | 0 | 2 | 3.0 | 3 | 7.9 |
| 2 | 0 | 0 | 1 | 1.5 | 3 | 7.9 |
| 3 | 0 | 0 | 5 | 7.5 | 12 | 31.6 |
| 4 | 0 | 0 | 8 | 11.9 | 4 | 10.5 |
| 5 | 1 | 1.4 | 18 | 26.9 | 4 | 10.5 |
| 6 | 1 | 1.4 | 15 | 22.4 | 5 | 13.2 |
| 7 | 4 | 5.6 | 6 | 9.0 | 4 | 10.5 |
| 8 | 5 | 6.9 | 4 | 6.0 | 0 | 0 |
| 9 | 10 | 13.9 | 5 | 7.5 | 2 | 5.3 |
| 10 | 7 | 9.7 | 2 | 3.0 | 1 | 2.6 |
| 11 | 15 | 20.8 | 0 | 0 | 0 | 0 |
| 12 | 12 | 16.7 | 1 | 1.5 | 0 | 0 |
| 13 | 11 | 15.3 | 0 | 0 | 0 | 0 |
| 14 | 5 | 6.9 | 0 | 0 | 0 | 0 |
| 15 | 1 | 1.4 | 0 | 0 | 0 | 0 |
| 16 | 0 | 0 | 0 | 0 | 0 | 0 |
| 17 | 0 | 0 | 0 | 0 | 0 | 0 |
| 18 | 0 | 0 | 0 | 0 | 0 | 0 |
| 19 | 0 | 0 | 0 | 0 | 0 | 0 |
| **Total number HCPs above the cut-off** | 17 | 23.6 | 8 | 12.0 | 3 | 7.9 |
| **Total** | 72 | 100 | 67 | 100 | 38 | 100 |

Abbreviation: HCP: healthcare personnel

The thick line represents the cut-off limit for competence in Ms. Olsen's test.
